# Supplementary material for: Oxygen and Pt(II) self-generating conjugate for synergistic photo-chemo therapy of hypoxic tumor
Source: Nat Commun. 2018 May 24;9:2053. doi: 10.1038/s41467-018-04318-1 (PMC5967320; doi:10.1038/s41467-018-04318-1)
Supplement: Supplementary file 1 — Supplementary Information [file 41467_2018_4318_MOESM1_ESM.pdf]

Supplementary Information

Oxygen and Pt(II) Self-generating Conjugate for Synergistic Photo-Chemo Therapy of Hypoxic Tumor

Shuting Xu, Xinyuan Zhu, Chuan Zhang, Wei Huang, Yongfeng Zhou, Deyue Yan*

School of Chemistry and Chemical Engineering, State Key Laboratory of Metal Matrix Composites, Shanghai key laboratory of electrical insulation and thermal ageing, Shanghai Jiao Tong University, 800 Dongchuan Road, Shanghai 200240, P. R. China

Corresponding author. E-mail addresses: dyyan@sjtu.edu.cn

Supplementary Methods

Synthesis of *cis,trans,cis*-$[Pt(N_3)_2(OH)_2(NH_3)_2]$

Cisplatin (1 equiv) was suspended in water. $AgNO_3$ (2 equiv) was added and stirred in the dark at room temperature for 24 h. Then the AgCl precipitate was removed by centrifugation. The clear solution was added with NaN_3 and then stirred for another 4h. The white precipitate was filtered and dried. H_2O_2 (40 equiv) was added to the suspension of *cis*-$[Pt(N_3)_2(NH_3)_2]$ in water which was under stirred for 4 h in the dark. The product was isolated by centrifugation to get the yellow powder.

Synthesis of *cis,trans,cis*-$[Pt(N_3)_2(OH)(NH_3)_2(O_2CCH_2CH_2COOH)]$

0.35 g of *cis,trans,cis*-$[Pt(N_3)_2(NH_3)_2(OH)_2]$ (1 equiv) and dry DMSO (20 mL) were added to the reaction vessel to get yellow clear solution. Succinic anhydride (1 equiv) was added to the above solution and stirred for 24 h. The resulting solution was dried through vacuum rotary evaporation to leave dark yellow oil, which was then dissolved in 5 ml methanol. Addition of large excess ether precipitated a yellow solid that was isolated and dried.

Conjugation of *cis,trans,cis*-$[Pt(N_3)_2(OH)(NH_3)_2(O_2CCH_2CH_2COOH)]$ onto PEG

cis,trans,cis-$[Pt(N_3)_2(OH)(NH_3)_2(O_2CCH_2CH_2COOH)]$(Pt(IV)-COOH) (0.1 mmol) was dissolved in 2 ml dry DMSO. EDC (0.15 mmol) and NHS (0.15 mmol) were added and stirred overnight protected from light. Following, the resulting solution was added dropwise slowly into NH_2-PEG-NH_2 (1 mmol) in DMSO and stirred for another 48 h. The above solution was dialyzed against DMSO (molecular weight cutoff: 1000 Da) and water (molecular weight cutoff: 2000 Da) to remove unreacted EDC and NHS within a dialysis bag. The aqueous solution of the NH_2-PEG-Pt(IV) conjugate was then lyophilized for use.

Conjugation of Ce6 onto PEG-Pt(IV)

Ce6-PEG-Pt(IV) was synthesized similar to the above. Briefly, 0.1 mmol of Ce6, EDC (0.2 mmol) and NHS (0.2 mmol) were dissolved in dry DMSO and stirred overnight. NH_2-PEG-Pt(IV) in DMSO was then added dropwise into the solution above. After two days, the above solution was dialyzed against DMSO and water within a dialysis bag (molecular weight cutoff: 2000 Da). The aqueous solution was then lyophilized for use.

Preparation of control groups: PEG-Pt(IV) and PEG-Ce6

For synthesis of PEG-Pt(IV), 67.1 mg of Pt(IV)-COOH (0.15 mmol) was dissolved in 2 ml dry DMSO. EDC (0.2 mmol) and NHS (0.2 mmol) were added and stirred overnight protected from light. Following, the resulting solution was added dropwise slowly into PEG-NH_2 (200 mg, 0.1 mmol) in DMSO and stirred for another 48 h. The above solution was dialyzed against DMSO and water to remove unreacted Pt(IV)-COOH, EDC and NHS within a dialysis bag (molecular weight cutoff: 2000 Da). The aqueous solution of the PEG-Pt(IV) conjugate was then lyophilized for use. The synthesis of PEG-Ce6 was similar to PEG-Pt(IV), in which Ce6 was substituted for Pt(IV)-COOH.

Preparation of UCNPs loaded nanoparticles

For preparing UCNPs loaded Ce6-PEG-Pt(IV) nanoparticles, we used a single-emulsion method. In short, 4.5 mg of Ce6-PEG-Pt(IV) dissolved in 1.5 ml of water and then 50 μL UCNPs in chloroform was added. The mixture was ultrasonicated for at 450 W (Sonics & Materials, Newtown, CT) for 2 min at 0 ℃ to form an oil-in-water emulsion. The additional chloroform was removed by rotary evaporation. As the control, UCNPs loaded PEG-Pt(IV) and PEG-Ce6 nanoparticles were synthesized with the similar method by replacing Ce6-PEG-Pt(IV) by PEG-Pt(IV) and PEG-Ce6.

Preparation of Cy5.5-UCPP nanoparticles

In a typical procedure for the preparation of Cy5.5-UCPP nanoparticles: 3.5 mg UCPP was dissolved in 1 mL of anhydrous DMSO, followed by adding a 0.1 mL of Cy5.5-NHS ester solution (1 mg mL^{-1}) in anhydrous DMSO and stirred at room temperature overnight. Then the mixture was slowly added into 3 mL of deionized water and stirred slightly for another 10 min. Subsequently, the solution was dialyzed against deionized water for 16 h (molecular weight cutoff = 1,000 g mol^{-1}) and the deionized water was exchanged for 4 times. In order to determine the amount of Cy5.5, the Cy5.5-UCPP nanoparticle solution was lyophilized and then dissolved in DMSO again. The Cy5.5 content in UCPP nanoparticles was determined by the absorbance at 680 nm measured using an UV/Vis spectrophotometer.

Cell culture

L929 cells (a mouse fibroblast cell line) and HeLa cells (a human cervical cancer line) were cultured in Dulbecco's Modified Eagle's medium (DMEM) supplied with 10% FBS (fetal bovine serum) and antibiotics (50 units mL^{-1} penicillin and 50 units mL^{-1} streptomycin) at 37 °C under a humidified atmosphere containing 5% CO_2. These cells line were obtained from Shanghai Cell Bank, Chinese Academy of Sciences (CAS).

Pharmacokinetic studies

SD rats (about 160 g) were chosen to study the pharmacokinetics of UCPP and free CDDP. Rats were injected with UCPP and free CDDP at a dose of 1 mg cisplatin per kg body weight through tail vein (n = 4). Blood samples (0.5 mL) were obtained from the retro-orbital plexus of eyes at different time points (15 min, 30 min, 1 h, 2 h, 4 h, 8 h, 12 h, and 24 h) after injection. Platinum concentration in the samples was examined by ICP-MS.

Biodistribution of UCPP

The HeLa tumor-bearing mice were caudal vein injection with CDDP (1 mg per kg body weight) and UCPP (10 mg per kg, 1 mg cisplatin per kg body weight). Mice were dissected at 2 h, 8 h, 12 h and 24 h (n = 3 at each time point) after drug administration, then heart, liver, spleen, lung, kidney and tumor were collected. The content of platinum in these tissues was measured by ICP-MS after nitrolysis.

***In vivo* and *ex vivo* optical imaging**

HeLa tumor-bearing mice were tail vein injected with free Cy5.5 and Cy5.5-loaded UCPP owned similar absorption intensity. The fluorescence distribution was measured at 2, 4, 6, 8, 12 and 24 h using an *in vivo* and *ex vivo* imaging system with excitation at 690 nm and emission at 700 nm. After 24 h, mice were sacrificed, and then heart, liver, spleen, lung, kidney and tumor were collected for *ex vivo* fluorescence imaging.

H&E staining and PCNA expression

HeLa tumor-bearing mice (~100 mm^3) were tail vein injected with PBS, UPP, UPC and UCPP. Four hours after the injection, mice were received light irradiation at the tumor sites. After 24 h post irradiation, the tumor sections were analyzed by H&E stain and PCNA expression. Anti-PCNA (clone PC10; ab29; Abcam) was applied to the sections at dilutions of 1:5000.

Statistical analysis

Quantitative data were expressed as mean ± SD. P values were calculated by two-tailed Student's t-test (***p < 0.001, **p < 0.01, or *p < 0.05). P < 0.05 was considered statistically significant. All statistical analyses were carried out using GraphPad Prism Software (Version 6.0, GrapPad Software, San Diego, CA).

Supplementary Figures

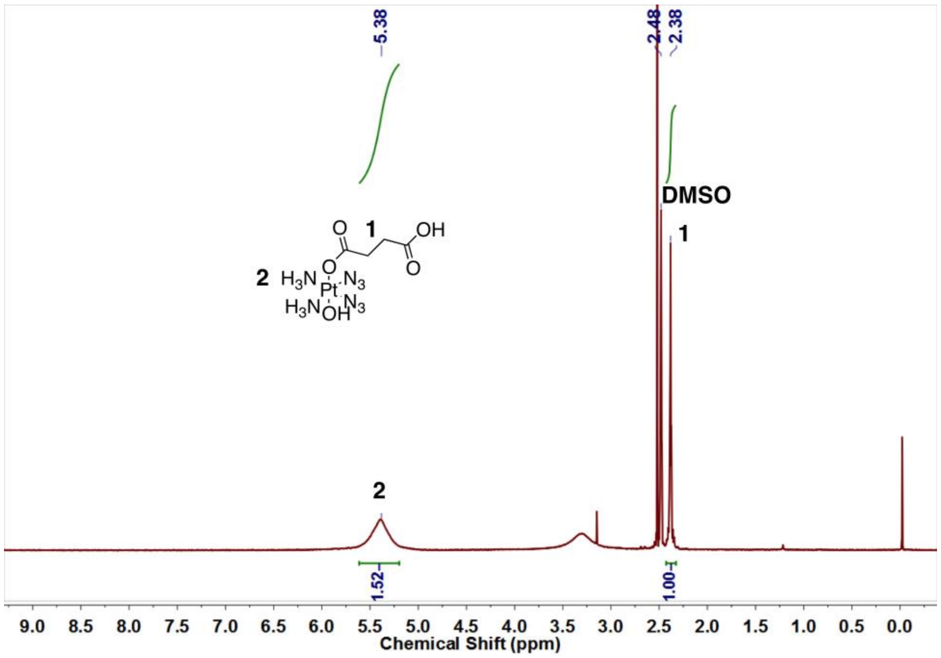

Supplementary Figure 1: ^{1}H NMR spectrum of Pt(IV) prodrug.

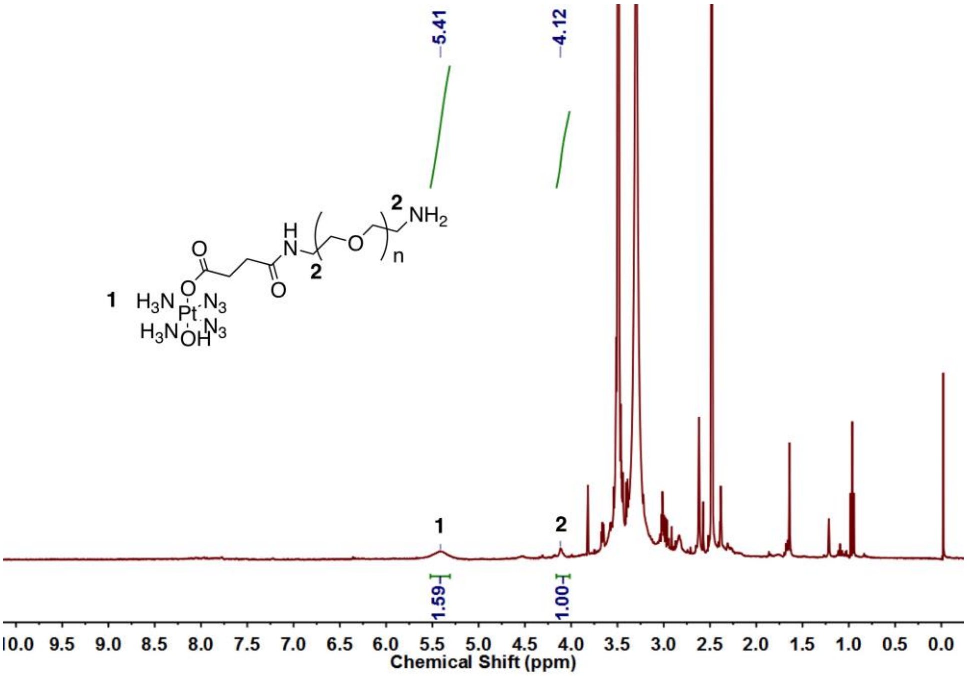

Supplementary Figure 2: ^{1}H NMR spectrum of NH_2-PEG-Pt(IV).

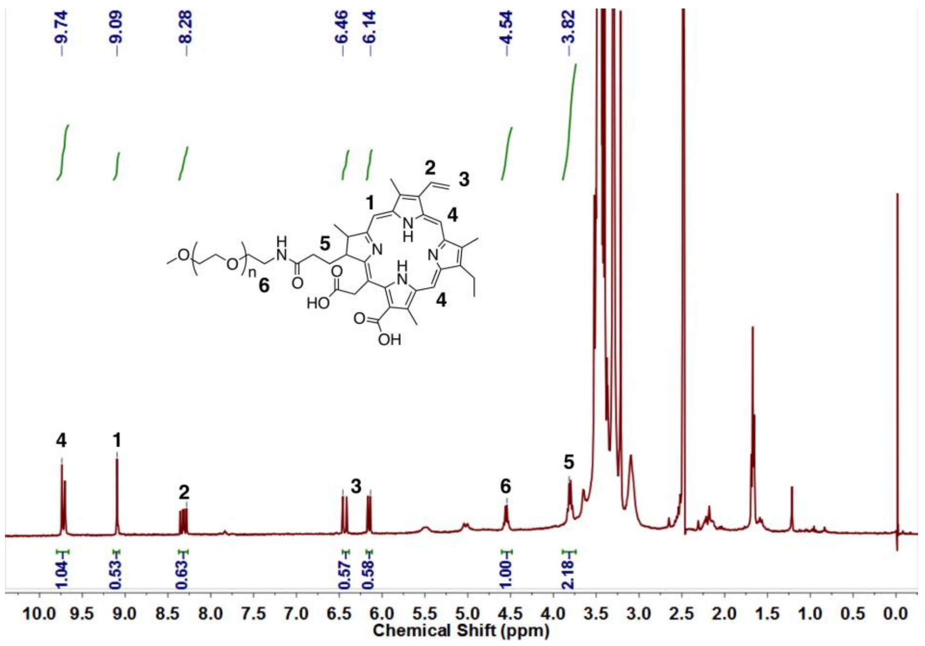

Supplementary Figure 3: ^{1}H NMR spectrum of control group PEG-Ce6.

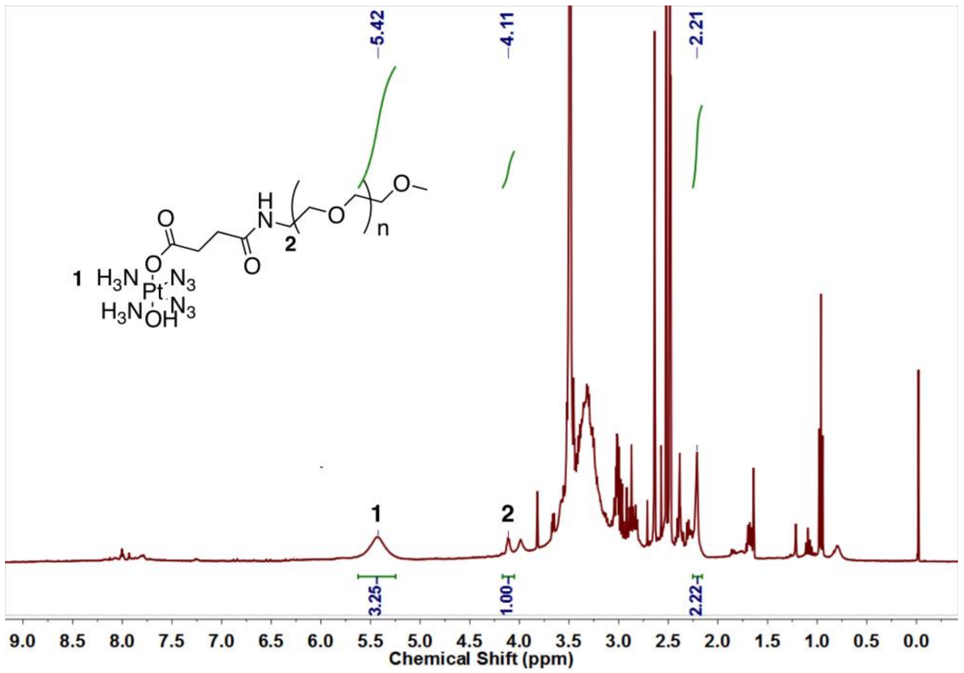

Supplementary Figure 4: ^{1}H NMR spectrum of PEG-Pt(IV).

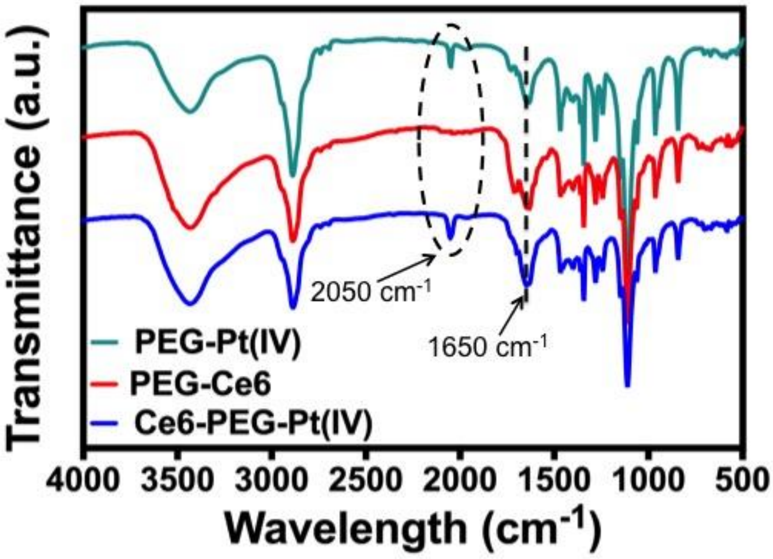

Supplementary Figure 5: FTIR spectra of PEG-Pt(IV), PEG-Ce6 and Ce6-PEG-Pt(IV).

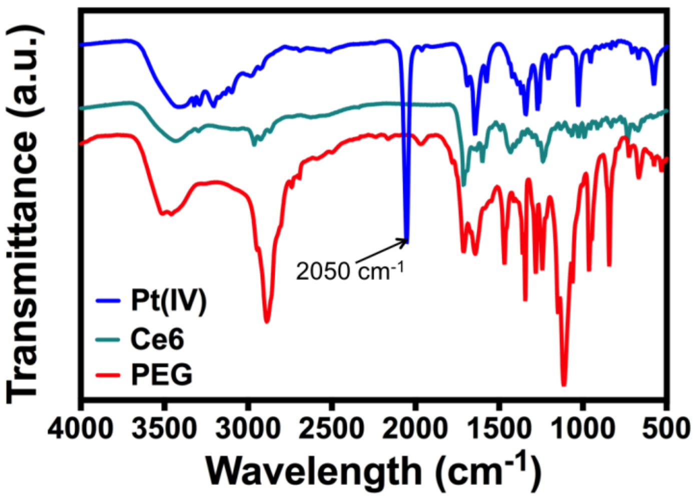

Supplementary Figure 6: FTIR spectra of Pt(IV), Ce6 and PEG.

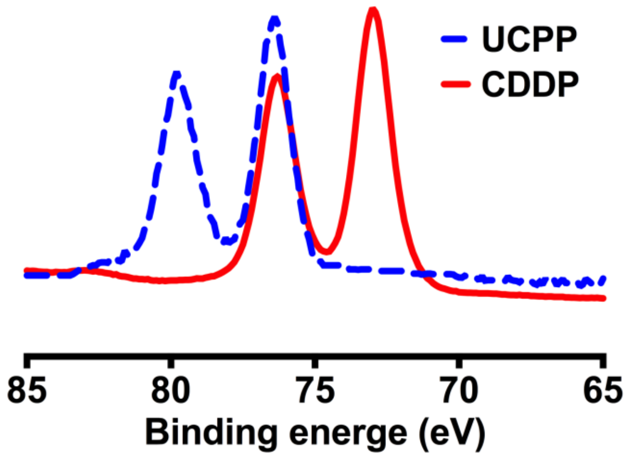

Supplementary Figure 7: High-resolution XPS spectra (Pt 4f) of CDDP and UCPP.

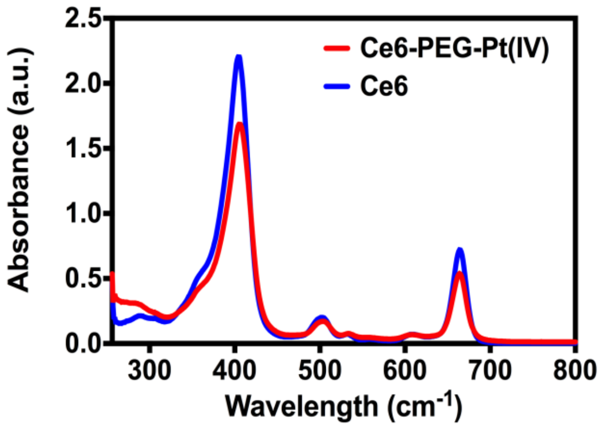

Supplementary Figure 8: UV/Vis spectra of Ce6-PEG-Pt(IV) and Ce6 in DMSO.

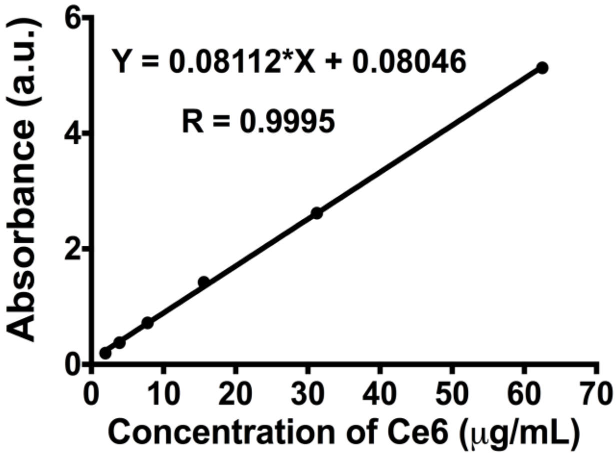

Supplementary Figure 9: Standard curve of Ce6.

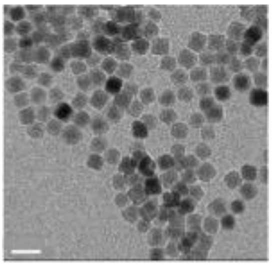

Supplementary Figure 10: TEM image of UCNPs, Scale bar = 20 nm.

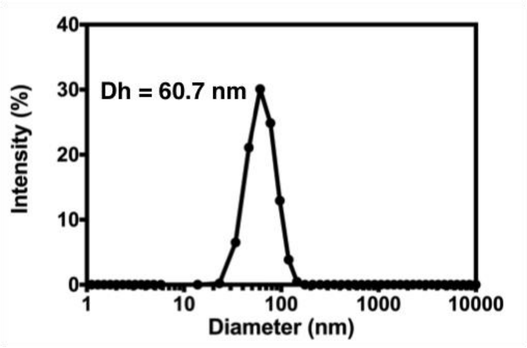

Supplementary Figure 11: Hydrodynamic size distribution of UCPP.

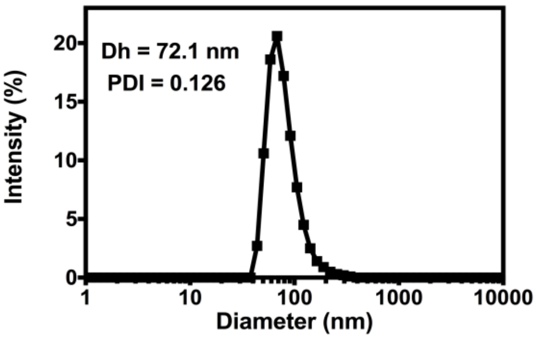

Supplementary Figure 12: DLS measurement of Cy5.5-UCPP.

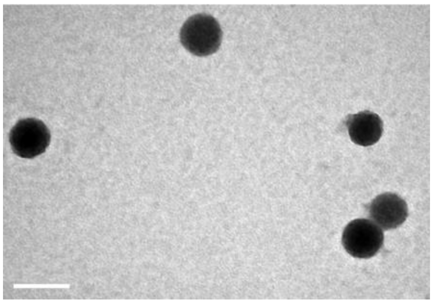

Supplementary Figure 13: The TEM image of Cy5.5-UCPP (Scale bar is 100 nm).

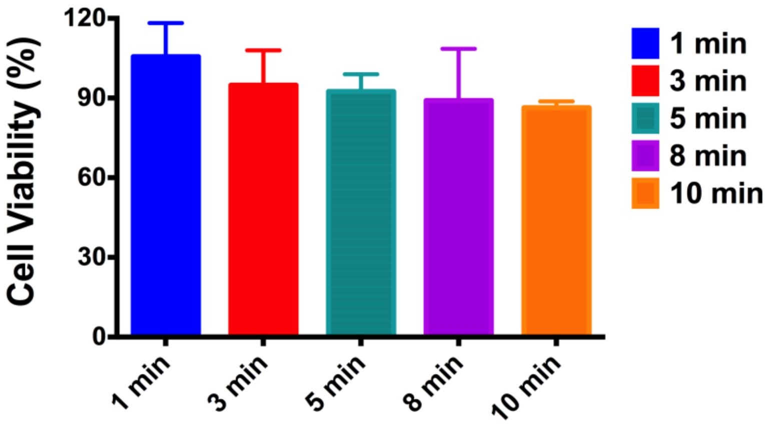

Supplementary Figure 14: Cell viability after being irradiated under NIR light for different irradiation times. Data were shown as mean ± S.D. (n = 5).

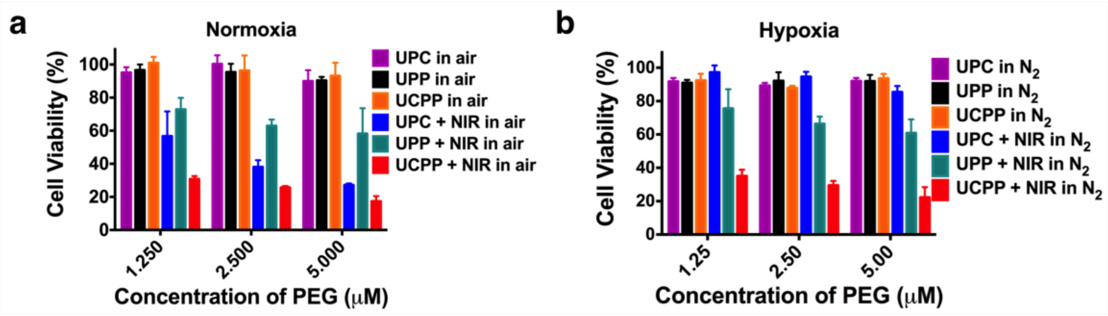

Supplementary Figure 15: *In vitro* cytotoxicity. (**a**) Relative viabilities of HeLa cells after incubation with various concentrations of UPC, UPP or UCPP with or without light irradiation in normoxic environment for 48 h. (**b**) Relative viabilities of HeLa cells after incubation with various concentrations of UPC, UPP or UCPP with or without light irradiation (5 min per every well) in hypoxic environment for 48 h. Data were shown as mean ± S.D. (n = 6).

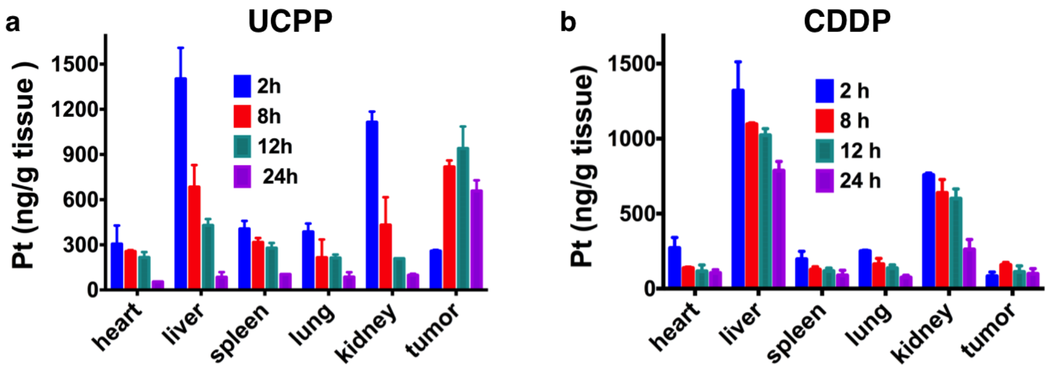

Supplementary Figure 16: The biodistribution of UCPP and free cisplatin at 2 h, 8 h, 12 h and 24 h after systemic administration based on ICP-MS analysis (data expressed as nanogram platinum per gram of tissue (Pt (ng per g tissue))). Data were shown as mean ± S.D. (n = 3).

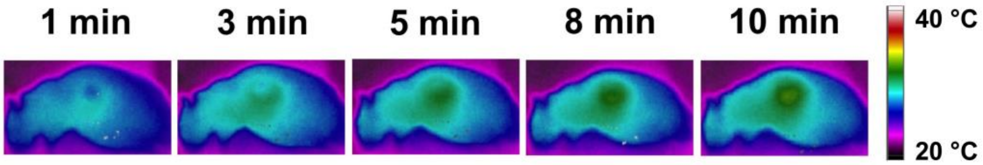

Supplementary Figure 17: The temperature of tumor site recorded by an IR camera under NIR irradiation for different times.

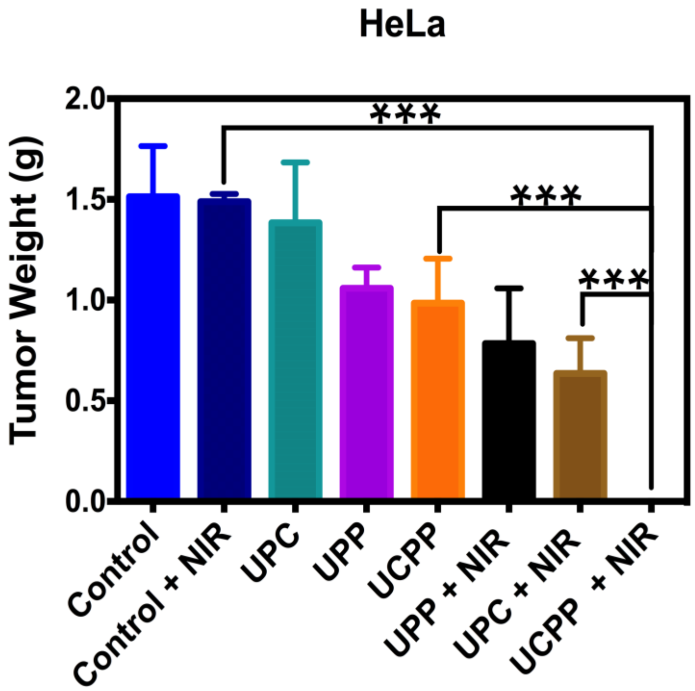

Supplementary Figure 18: Tumor weight of HeLa tumor-bearing mice after treating with PBS (blue), PBS + NIR (deep blue), UPC (cyan), UPP (purple), UCPP (orange), UPP + NIR (black), UPC + NIR (brown), UCPP + NIR (red). Data were shown as mean ± S.D. (n = 5). P values by comparing the UCPP + NIR group with other control groups were calculated by two-tailed Student's t-test (***$p < 0.001$, **$p < 0.01$, or *$p < 0.05$).

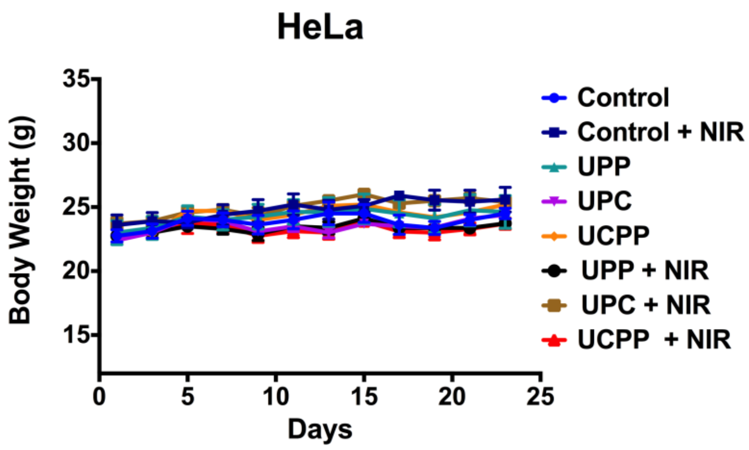

Supplementary Figure 19: Body weight of HeLa tumor-bearing mice after treating with PBS (blue), PBS + NIR (deep blue), UPC (cyan), UPP (purple), UCPP (orange), UPP + NIR (black), UPC + NIR (brown), UCPP + NIR (red). Data were shown as mean ± S.D. (n = 5).

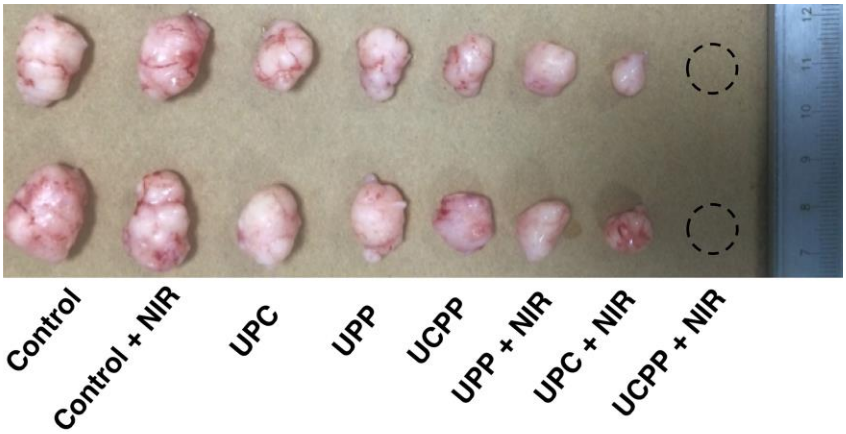

Supplementary Figure 20: Images of striped HeLa tumors after the treatment (healed tumors were delineated with imaginary black circle).

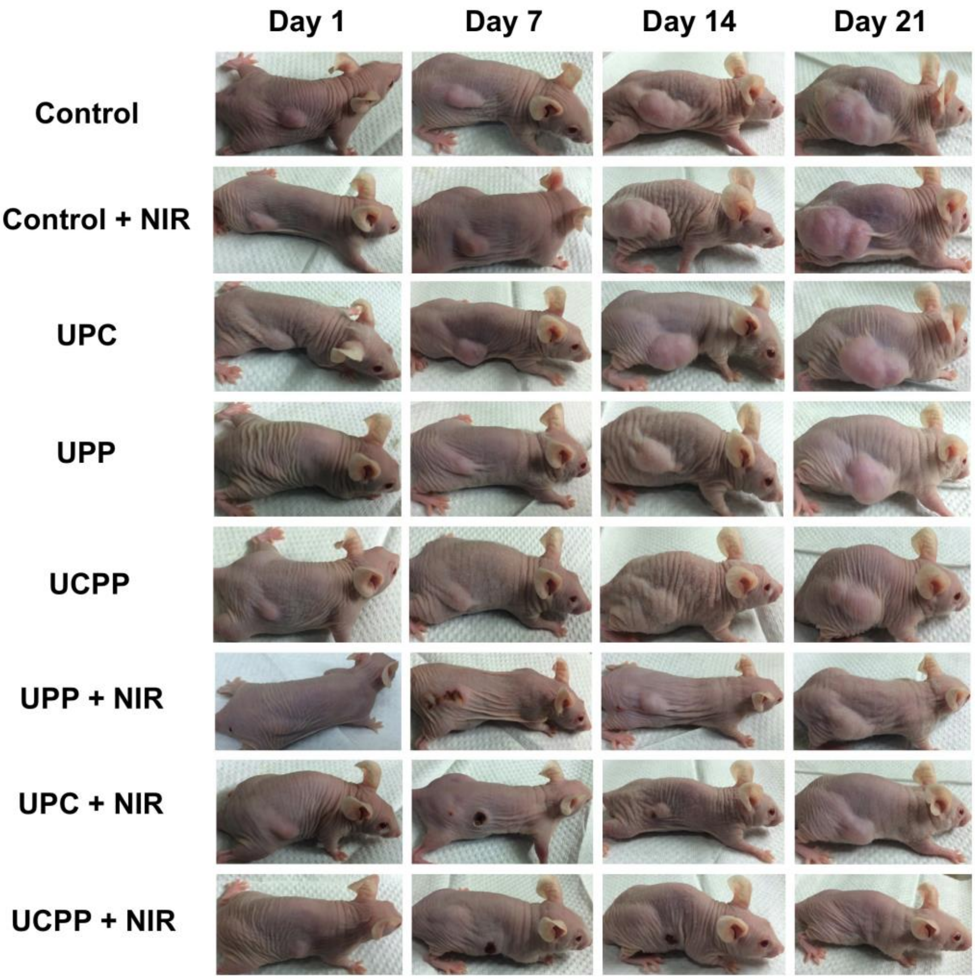

Supplementary Figure 21: Photos of HeLa tumor-bearing mice treated with PBS, PBS and NIR irradiation, UPC, UPP, UCPP, UPP and NIR irradiation, UPC and NIR irradiation, UCPP and NIR irradiation during 23-day treatment.

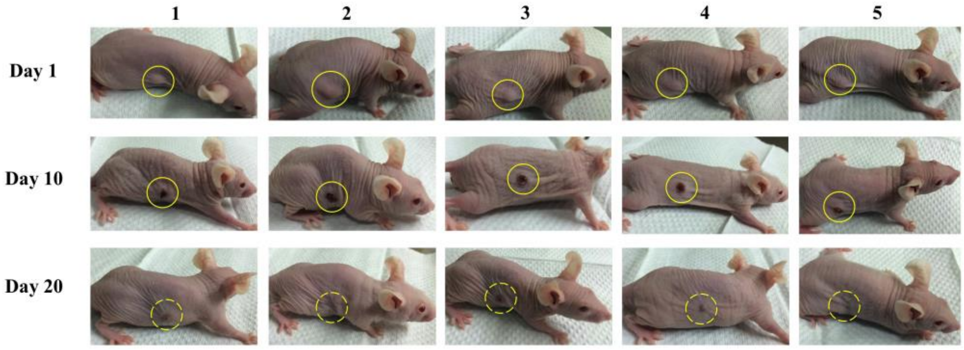

Supplementary Figure 22: The representative photos of five HeLa tumor-bearing mice treated with UCPP + NIR during 23-day evaluation period. Tumors were delineated with full yellow circle, while healed tumors were delineated with imaginary yellow circle.

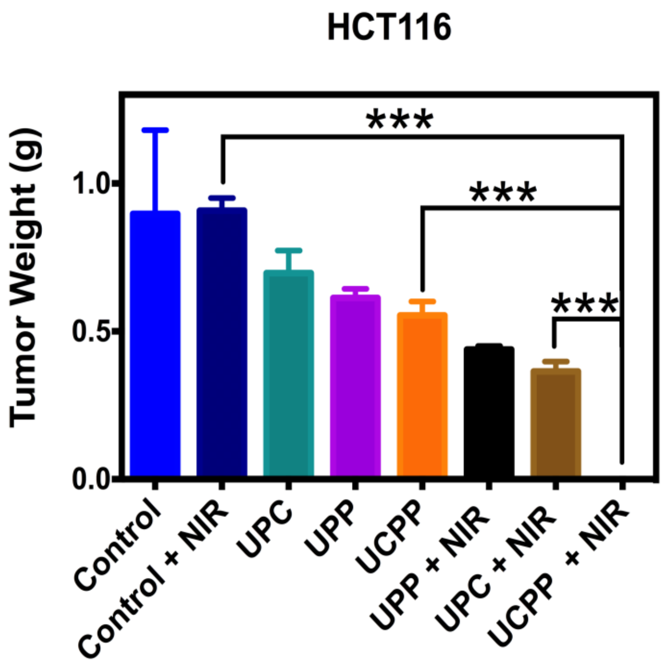

Supplementary Figure 23: Tumor weight of HCT116 tumor-bearing mice after treating with PBS (blue), PBS + NIR (deep blue), UPC (cyan), UPP (purple), UCPP (orange), UPP + NIR (black), UPC + NIR (brown), UCPP + NIR (red). Data were shown as mean ± S.D. (n = 5). P values by comparing the UCPP + NIR group with other control groups were calculated by two-tailed Student's t-test (***p < 0.001, **p < 0.01, or *p < 0.05).

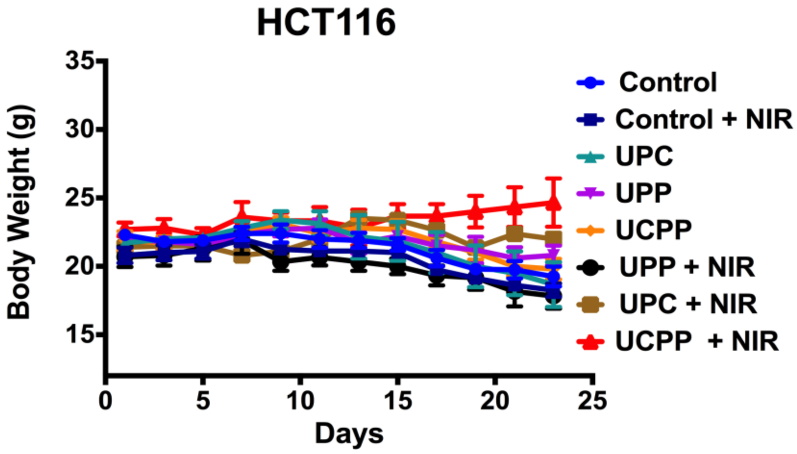

Supplementary Figure 24: Body weight of HCT116 tumor-bearing mice after treating with PBS (blue), PBS + NIR (deep blue), UPC (cyan), UPP (purple), UCPP (orange), UPP + NIR (black), UPC + NIR (brown), UCPP + NIR (red). Data were shown as mean ± S.D. (n = 5).

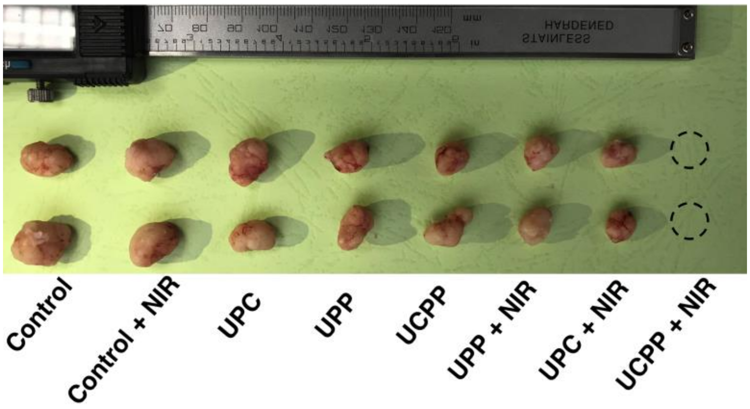

Supplementary Figure 25: Images of striped HCT116 tumors after the treatment (healed tumors were delineated with imaginary black circle).

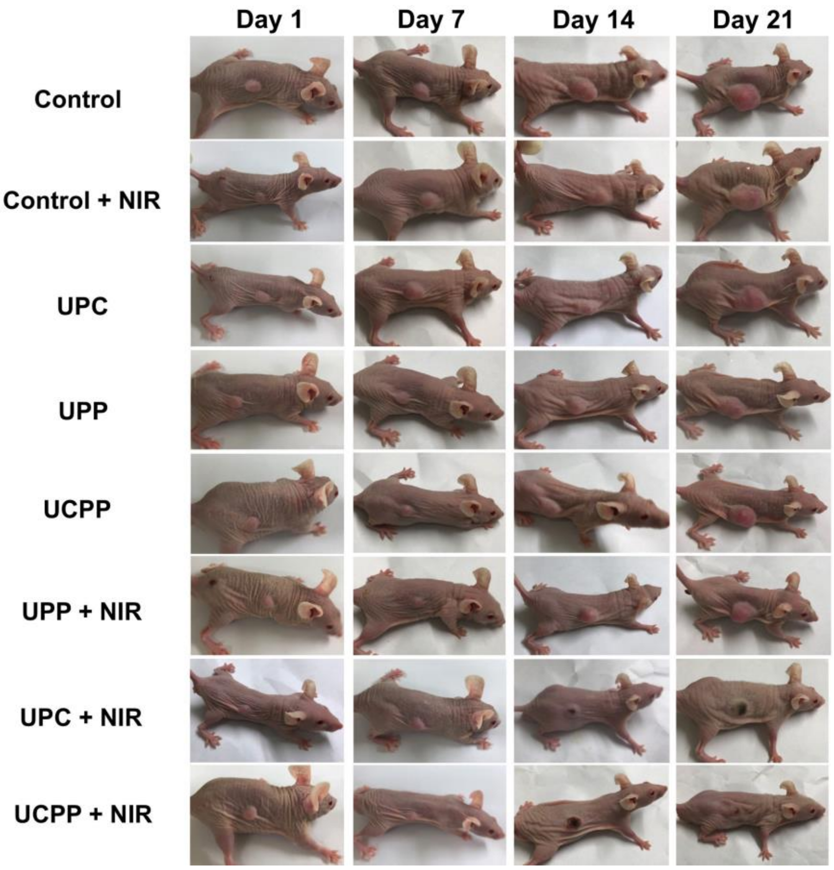

Supplementary Figure 26: Photos of HCT116 tumor-bearing mice treated with PBS, PBS and NIR irradiation, UPC, UPP, UCPP, UPP and NIR irradiation, UPC and NIR irradiation, UCPP and NIR irradiation during 23-day treatment.

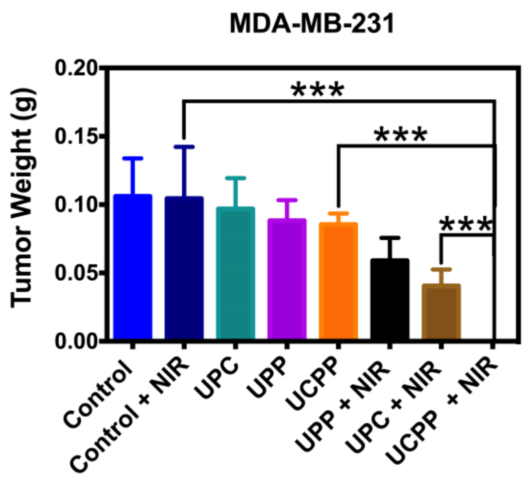

Supplementary Figure 27: Tumor weight of MDA-MB-231 tumor-bearing mice after treated with PBS (blue), PBS + NIR (deep blue), UPC (cyan), UPP (purple), UCPP (orange), UPP + NIR (black), UPC + NIR (brown), UCPP + NIR (red). Data were shown as mean ± S.D. (n = 5). P values by comparing the UCPP + NIR group with other control groups were calculated by two-tailed Student's t-test (***p < 0.001, **p < 0.01, or *p < 0.05).

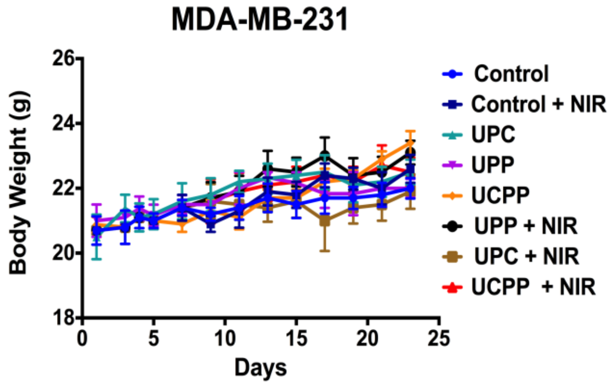

Supplementary Figure 28: Body weight of MDA-MB-231 tumor-bearing mice after treated with PBS (blue), PBS + NIR (deep blue), UPC (cyan), UPP (purple), UCPP (orange), UPP + NIR (black), UPC + NIR (brown), UCPP + NIR (red). Data were shown as mean ± S.D. (n = 5).

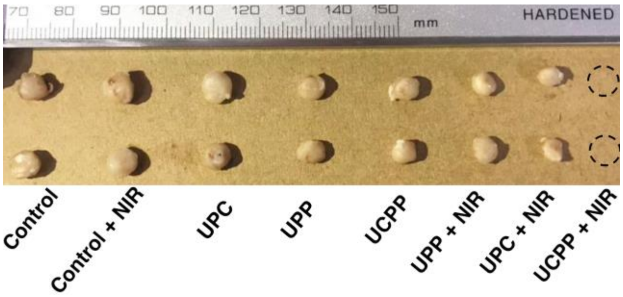

Supplementary Figure 29: Images of striped MDA-MB-231 tumors after the treatment (healed tumors were delineated with imaginary black circle).

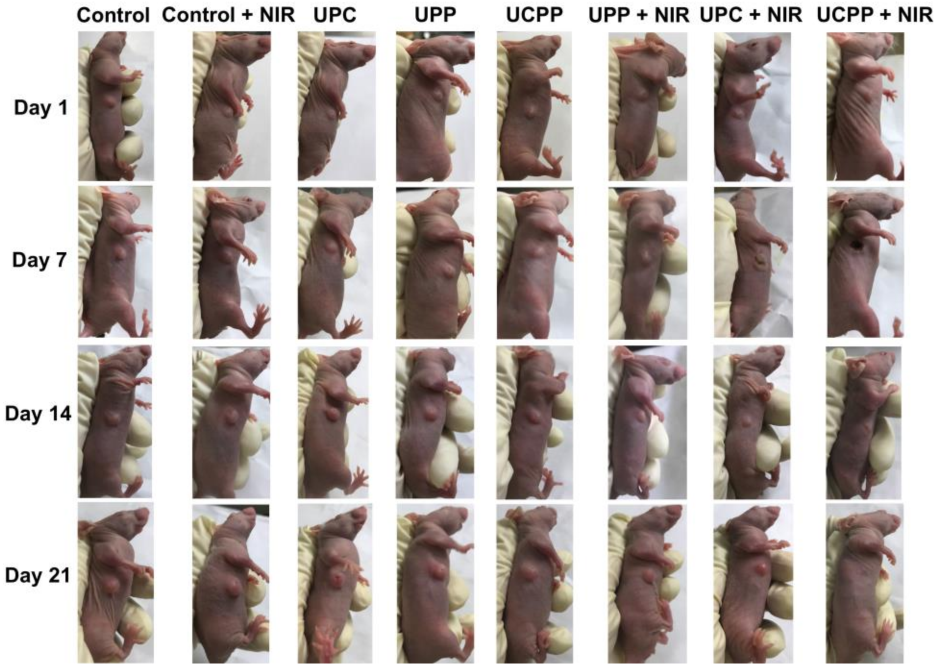

Supplementary Figure 30: Photos of MDA-MB-231 tumor-bearing mice treated with PBS, PBS and NIR irradiation, UPC, UPP, UCPP, UPP and NIR irradiation, UPC and NIR irradiation, UCPP and NIR irradiation during 23-day treatment.

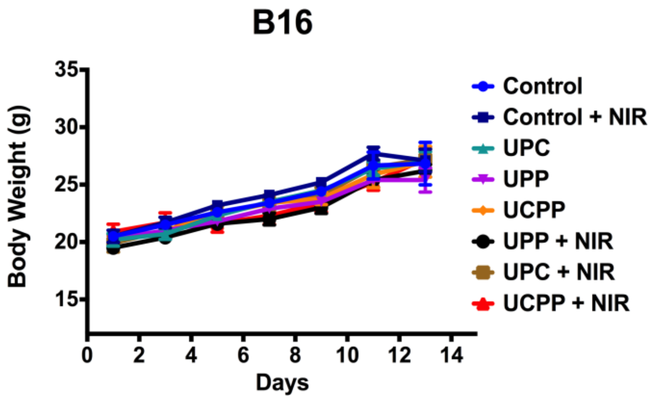

Supplementary Figure 31: Body weight of B16 tumor-bearing mice after treating with PBS (blue), PBS + NIR (deep blue), UPC (cyan), UPP (purple), UCPP (orange), UPP + NIR (black), UPC + NIR (brown), UCPP + NIR (red). Data were shown as mean ± S.D. (n = 5).

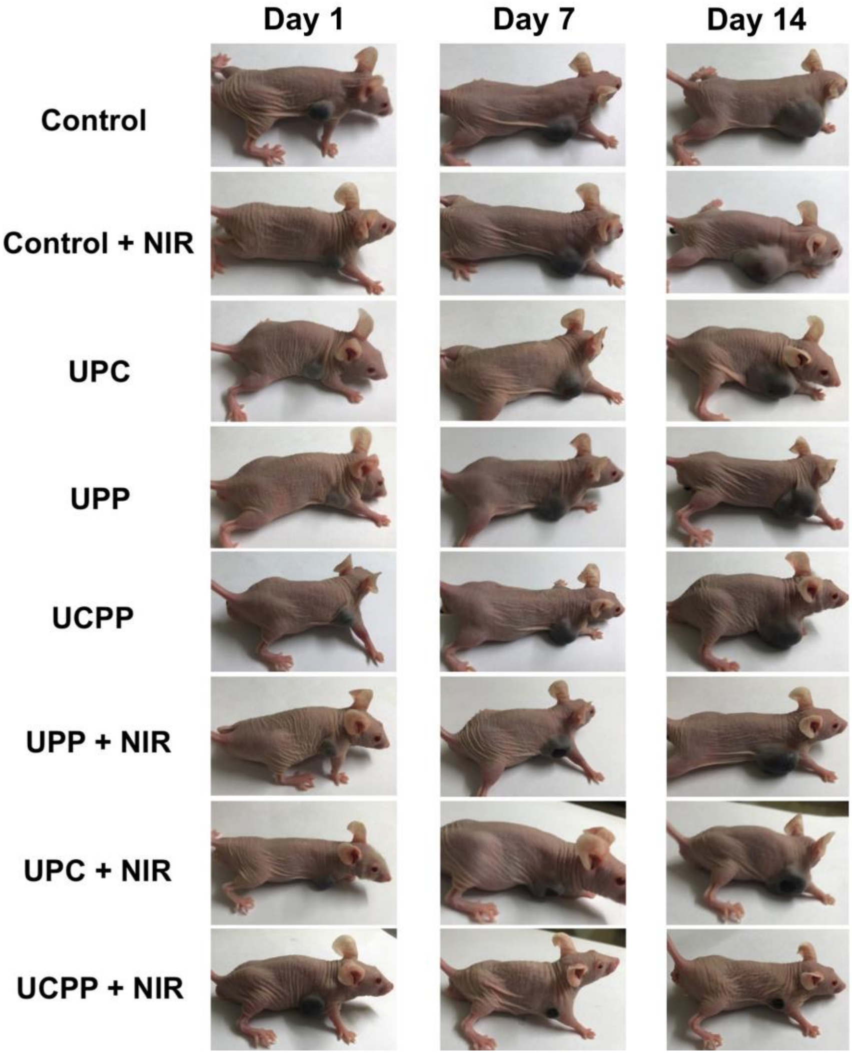

Supplementary Figure 32: Photos of B16 tumor-bearing mice treated with PBS, PBS and NIR irradiation, UPC, UPP, UCPP, UPP and NIR irradiation, UPC and NIR irradiation, UCPP and NIR irradiation during 14-day treatment.

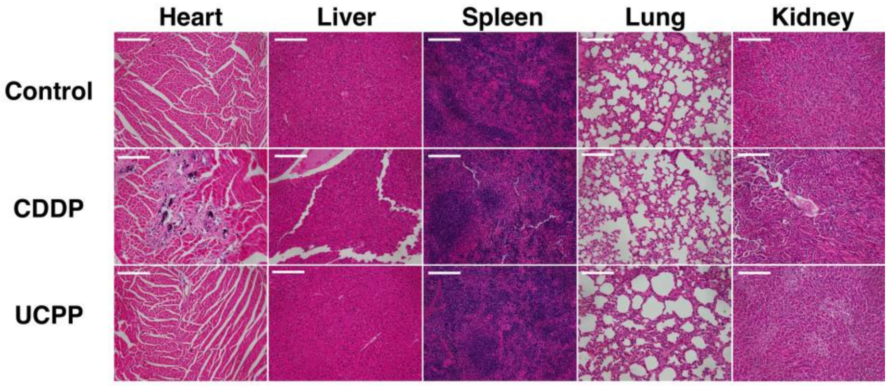

Supplementary Figure 33: H&E stain of organs (HeLa tumor-bearing mice) after administrated with CDDP and UCPP irradiation (magnification × 200). Scale bar = 100 μm.

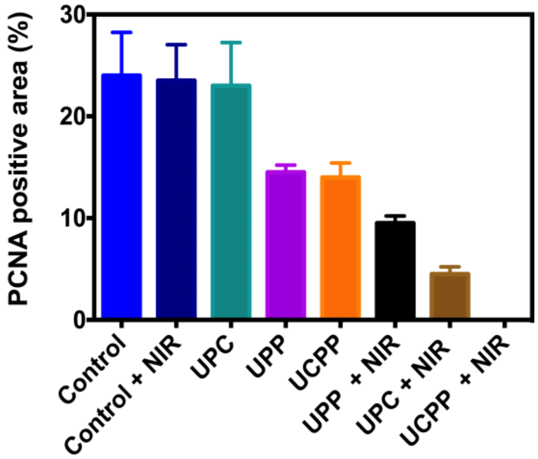

Supplementary Figure 34: The relative PCNA positive areas were recorded using the ImageJ software. Data were shown as mean ± S.D. (n = 5).

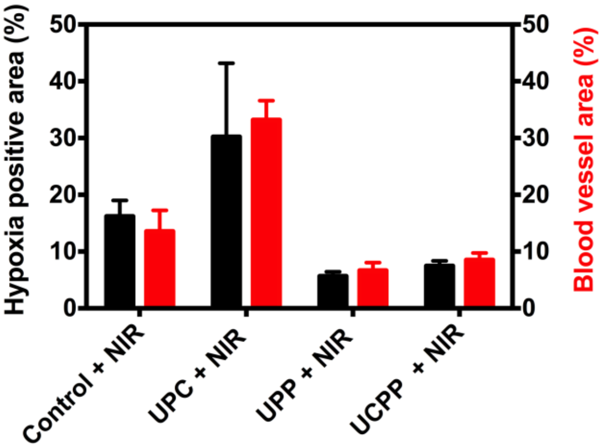

Supplementary Figure 35: The relative hypoxia positive areas and blood vessel densities were recorded using the ImageJ software. Data were shown as mean ± S.D. (n = 5).

Supplementary Discussion 1. Characterization of CPP, intermediate products and control groups

As shown in **Supplementary Figure 1**, the integral ratio of the peak at 2.38 ppm (1) and 5.38 ppm (2) was 1:1.5, indicating the successful synthesis of Pt(IV) prodrug.

As shown in **Supplementary Figure 2**, the integral ratio of the peak at 5.41 ppm (1) and 4.12 ppm (2) was 1.5:1, indicating the successful synthesis of intermediate product NH_2-PEG-Pt(IV).

As shown in **Supplementary Figure 3**, the integral ratio of the peak at 9.74 ppm (4) and 4.54 ppm (6) was 1:1, indicating that control group PEG-Ce6 was synthesized successfully with a ratio of 1:1.

As shown in **Supplementary Figure 4**, the integral ratio of the peak at 5.42 ppm (1) and 4.11 ppm (2) was 3:1, indicating the successful synthesis of control group PEG-Pt(IV).

In the FTIR spectra (**Supplementary Figure 5**) of PEG-Pt(IV) and Ce6-PEG-Pt(IV), the azido absorption band at 2050 cm^{-1} can be observed, indicating that Pt(IV) was conjugated in polymer Ce6-PEG-Pt(IV). A strong C=O stretching absorption band at 1650 cm^{-1} attributes to amide appeared in the spectra, showing that Pt(IV) or Ce6 was conjugated with PEG.

In the FTIR spectra (**Supplementary Figure 6**) of Pt(IV), the azido absorption band at 2050 cm^{-1} can be observed.

Based on the XPS spectra (**Supplementary Figure 7**), Pt 4f in cisplatin exhibited characteristic binding energy at 72.9 eV Pt (4f7/2) and 76.0 eV Pt (4f5/2) respectively, while the binding energy of Pt in UCPP were shifted to 79.7 eV (Pt 4f5/2) and 76.3 eV (Pt 4f7/2). This result indicating that Pt(IV) was conjugated with PEG successfully.

Compared to the UV/Vis absorption (**Supplementary Figure 8**) of free Ce6 at 401 nm, we observed a 5 nm red-shift in the absorption of Ce6-PEG-Pt(IV) at 406 nm. Furthermore, Ce6-PEG-Pt(IV) possessed the UV/Vis absorptions of Ce6. The UV/Vis results further confirmed the successful synthesis of CPP.

Supplementary Discussion 2. Standard curve of Ce6

As shown in **Supplementary Figure 9**, the standard curve was conducted by the relationship between the absorbance value at 660 nm and the concentration of Ce6. The concentration of Ce6 in 0.25 mg per mL CPP was 10.066 μg per mL calculating from the standard curve.

Supplementary Discussion 3. TEM image of UCNPs

The TEM image in **Supplementary Figure 10** showed the average size of pure UCNPs was 10 nm.

Supplementary Discussion 4. The morphology and size of Cy5.5-UCPP

Supplementary Figure 12 gave the size distribution of the Cy5.5-UCPP and their average size (Dh = 72.1 nm) and polydispersity index (PDI = 0.126). Both size and polydispersity index of Cy5.5-UCPP are very similar to those of UCPP without Cy5.5 (Supplementary Figure 11).

As shown in **Supplementary Figure 13**, the average size of Cy5.5-UCPP is about 54 nm, which is similar to that of UCPP without Cy5.5 (Fig. 2b).

Supplementary Discussion 5. Cell viabilities in different treatments

When we treated the cancer cells with NIR alone for different times, the cell viability was extremely high, indicating that NIR irradiation alone had little effect towards therapeutic effect (see **Supplementary Figure 14**).

As shown in **Supplementary Figure 15**, no matter in normoxic or hypoxic environment, UPC, UPP and UCPP showed negligible cytotoxicity in the dark. Once exposed to laser irradiation, UPC showed high toxicity towards HeLa cells in normoxic environment, while no significant cytotoxicity was observed in hypoxic condition. In contrast, UPP and UCPP showed efficient cytotoxicity in both normoxic and hypoxic environments under irradiation.

Supplementary Discussion 6. Tissue distribution studies of UCPP and CDDP

As displayed in **Supplementary Figure 16**, free CDDP mainly accumulated in kidney and liver, but the concentration of Pt at the tumor site was quite low. On the contrary, UCPP could gradually accumulate in the tumor tissue up to 24 h, while the concentrations of Pt in organs were rapidly eliminated.

Supplementary Discussion 7. Heating effect of NIR alone

As shown in **Supplementary Figure 17**, we studied the heating effect of 980 nm laser alone using infrared thermal camera. The result indicated that the temperature of mouse had little enhancement as time increasing when the tumor exposed to NIR alone.

Supplementary Discussion 8. System toxicity of UCPP

As shown in **Supplementary Figure 33**, free CDDP had cardiotoxicity, hepatotoxicity and nephrotoxicity, while UCPP showed little toxicity towards normal tissues, indicating low systematic toxicity of the UCPP nanoparticles.

Supplementary Discussion 9. Quantification of PCNA stain

As shown in the **Supplementary Figure 34**, the least number of positive tumor cells revealed in the PCNA expression were observed in the synergistic therapy group using UCPP, while the cells in other groups largely or partly remained their proliferation.

Supplementary Discussion 10. Quantification of HIF-1α and CD31 stains

Compared to the PBS group, UPC treated tumor slice displayed obviously enhanced hypoxia positive area and blood vessel area, indicating that UPC exaggerate the tumor hypoxia. On the contrary, after UPP and UCPP treatment, reduced areas of hypoxia positive and blood vessel were observed, which are attributed to the O_2-generation of Pt(IV) under irradiation (see **Supplementary Figure 35**).
